# Structural differences contributing to sex-specific associations between FN BMD and whole-bone strength for adult White women and men

**DOI:** 10.1093/jbmrpl/ziae013

**Published:** 2024-01-30

**Authors:** Karl J Jepsen, Erin M R Bigelow, Robert W Goulet, Bonnie T Nolan, Michael A Casden, Kathryn Kennedy, Samantha Hertz, Chandan Kadur, Gregory A Clines, Aleda M Leis, Carrie A Karvonen-Gutierrez, Todd L Bredbenner

**Affiliations:** Department of Orthopaedic Surgery, University of Michigan, Ann Arbor, MI 48109 United States; Department of Orthopaedic Surgery, University of Michigan, Ann Arbor, MI 48109 United States; Department of Orthopaedic Surgery, University of Michigan, Ann Arbor, MI 48109 United States; Department of Orthopaedic Surgery, University of Michigan, Ann Arbor, MI 48109 United States; Department of Orthopaedic Surgery, University of Michigan, Ann Arbor, MI 48109 United States; Biomedical Engineering, Marquette University, Milwaukee, WI 53201 United States; Department of Orthopaedic Surgery, University of Michigan, Ann Arbor, MI 48109 United States; Department of Orthopaedic Surgery, University of Michigan, Ann Arbor, MI 48109 United States; Biomedical Laboratory R&D, VA Medical Center, Ann Arbor, MI 48105 United States; Department of Orthopaedic Surgery, University of Michigan, Ann Arbor, MI 48109 United States; Department of Orthopaedic Surgery, University of Michigan, Ann Arbor, MI 48109 United States; Department of Orthopaedic Surgery, University of Michigan, Ann Arbor, MI 48109 United States; Department of Mechanical and Aerospace Engineering, University of Colorado Colorado Springs, Colorado Springs, CO 80918 United States

**Keywords:** aging, analysis/quantitation of bone (DXA), analysis/quantitation of bone (other—strength), diseases and disorders of bone (osteoporosis), orthopedics (biomechanics)

## Abstract

Hip areal BMD (aBMD) is widely used to identify individuals with increased fracture risk. Low aBMD indicates low strength, but this association differs by sex with men showing greater strength for a given aBMD than women. To better understand the structural basis giving rise to this sex-specific discrepancy, cadaveric proximal femurs from White female and male donors were imaged using nano-CT and loaded in a sideways fall configuration to assess strength. FN pseudoDXA images were generated to identify associations among structure, aBMD, and strength that differ by sex. Strength correlated significantly with pseudoDXA aBMD for females (*R*^2^ = 0.468, *P* < .001) and males (*R*^2^ = 0.393, *P* < .001), but the elevations (*y*-intercepts) of the linear regressions differed between sexes (*P* < .001). Male proximal femurs were 1045 N stronger than females for a given pseudoDXA aBMD. However, strength correlated with pseudoDXA BMC for females (*R*^2^ = 0.433, *P* < .001) and males (*R*^2^ = 0.443, *P* < .001) but without significant slope (*P* = .431) or elevation (*P* = .058) differences. Dividing pseudoDXA BMC by FN-width, total cross-sectional area, or FN-volume led to significantly different associations between strength and the size-adjusted BMC measures for women and men. Three structural differences were identified that differentially affected aBMD and strength for women and men: First, men had more bone mass per unit volume than women; second, different cross-sectional shapes resulted in larger proportions of bone mass orthogonal to the DXA image for men than women; and third, men and women had different proportions of cortical and trabecular bone relative to BMC. Thus, the proximal femurs of women were not smaller versions of men but were constructed in fundamentally different manners. Dividing BMC by a bone size measure was responsible for the sex-specific associations between hip aBMD and strength. Thus, a new approach for adjusting measures of bone mass for bone size and stature is warranted.

## Graphical Abstract

**Graphical Abstract:**
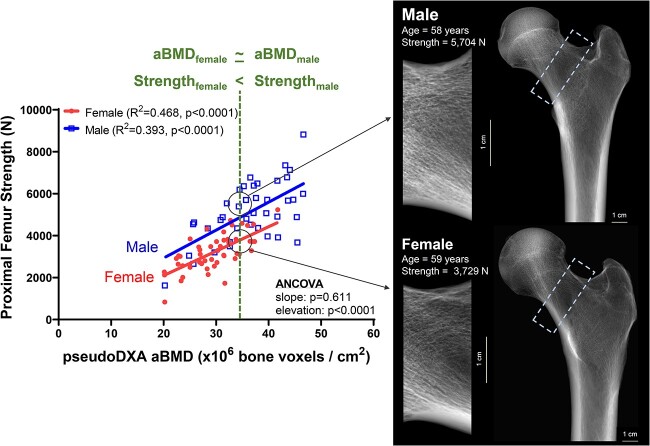

## Introduction

DXA is widely used clinically to identify women and men with low hip areal BMD (aBMD) who may be at increased risk of fracturing and therefore benefit from prophylactic treatment.^1^ Low FN aBMD is associated with increased hip fracture risk,^1^ which is generally thought to result from low bone strength.^2^ Although women and men show similar fracture risks at the same aBMD values,^3^^,^^4^ the association between FN aBMD and experimentally determined whole-bone strength shows a large sex-specific difference.^5–8^ Male proximal femurs are 800–1000 N stronger than women for a given FN aBMD.^7^ As a result of differences in aBMD–strength associations, it is necessary to consider the sex of the group used for aBMD comparisons when making treatment decisions based on aBMD.^9^^,^^10^

The goal of this study was to identify differences in bone structure that contribute to the sex-specific associations between aBMD and strength. Understanding discrepancies in the association between aBMD and strength is important given the prevalent clinical reliance on aBMD and growing interest in using aBMD as an endpoint in clinical trials.^11^ aBMD is calculated as BMC divided by the projected bone area. We hypothesize that the sex-specific associations arise from structural features that differ between women and men and that affect both the numerator (BMC) and the denominator (area) in the calculation of aBMD. On average, men have wider FNs with thicker cortices compared to women.^12–15^ Further, men have greater bone mass than women, independent of body stature^16^ and external bone size.^17^ Structural differences between women and men have generally been reported for the population average.^18^ The analyses to date have not revealed how the structural differences between women and men contribute to different aBMD–strength associations. To address this, we investigated how tissue distribution within a three-dimensional (3D) structure is projected onto a two-dimensional (2D) image and gives rise to BMC and area values measured by DXA. More specifically, we tested for differences in cross-sectional morphology that exist in 3D but that would be out of plane and not apparent from a 2D DXA image. We further tested how variation in BMC could arise from different proportions of cortical and trabecular bone. Finally, because our interests extend beyond the population average,^19^ we used regression-based analyses to systematically evaluate if the structural differences between women and men are consistent across the full range of data or whether these associations vary within each sex.

## Materials and methods

### Samples

Unfixed cadaveric proximal femurs from White female (*n* = 51; 24–89+ years of age) and male (*n* = 44; 18–89 years of age) donors were acquired from Science Care (Phoenix, AZ, USA), Anatomy Gifts Registry (Hanover, MD, USA), and the University of Michigan Anatomical Donations program (Ann Arbor, MI, USA). The samples had no known musculoskeletal pathologies. Human tissue use was approved by the Institutional Biosafety Committee (IBC), and this study was declared exempt by the Institutional Review Board (IRB). Proximal femur samples were generated by cutting the femoral shaft 16.5 cm distal to the superior aspect of the femoral head. The femoral shaft was embedded in a 5-cm square aluminum channel filled with acrylic resin (Ortho-Jet BCA, Lang Dental, Wheeling IL, USA) with the FN oriented at 15 degrees of internal rotation relative to the embedding block faces. Consistent sample orientation was enabled with the use of a custom-made alignment fixture. The strength and pseudoDXA aBMD parameters of 19 samples were reported previously for the validation study of the pseudoDXA images^20^ and are being used in this study to test for sex-specific associations.

### Nano-CT imaging

High-resolution 3D images were acquired for samples using a nano-CT system (nanotom-m, phoenix|x-ray, Wunstorf, Germany). Preventative maintenance was conducted biannually. The following image acquisition and reconstruction parameters were used for the samples: 27-μm voxel size, 110 kV, 200 μA, 0.762-mm aluminum filter.^21^ High-quality images were obtained by ensuring the images had the widest range in gray values possible and used ~80% of the dynamic range of the detector, by minimizing motion artifact using custom sample holders, using high-pass filters to reduce beam hardening, maintaining sample hydration, and using the same hydration fluid for all samples in the study. Some samples were imaged on a previous nano-CT system (nanotom-s) using similar acquisition parameters. Image volumes were reconstructed using datos|x software (GE Inspection Technologies, LP, Skaneateles, NY, USA). Each scan included a calibration phantom containing air, water, and a hydroxyapatite mimicker (1.69 mg/cc; Gammex, Middleton, WI, USA) to convert attenuation grayscale values to Hounsfield units, and intensity values associated with the phantom regions were monitored for consistency over time. Using our previously validated methods,^20^ each nano-CT volume was rotated 15 degrees to account for anteversion and an FN volume of interest (VOI) corresponding to a standard DXA ROI was generated using Dragonfly software (version 2021.1.0.977; Object Research Systems Inc, Montreal, Canada). The FN VOI width of 15 mm and its location adjacent to the greater trochanter corresponded to that of a Hologic scanner, as used for the validation study. Although any FN VOI location, orientation, and size could be extracted, we continued using the FN VOI consistent with that of the Hologic system for rigor and clinical relevance. The FN VOI extraction process had excellent reliability with an intraclass correlation coefficient (ICC) of 0.9997 (95% CI: 0.9987–0.9999) for area and 0.988 (95% CI: 0.9433–0.9987) for BMC. Sample-by-sample segmentation was conducted to minimize the impact of minor scan-to-scan variations in attenuation on outcome variables. Segmentation of bone-from-background and cortical-from-trabecular bone were conducted using two different validated fully convolutional neural networks (FCNNs), each with Dice coefficients of ~0.98. The cortical–trabecular boundary was defined in accordance with prior studies.^22^^,^^23^ Each segmented image was evaluated manually for thresholding quality and cortical–trabecular segmentation, and false-positive and false-negative voxels were manually corrected. For consistency, the same individual (E.M.R.B.) evaluated the accuracy of bone-to-background and cortical–trabecular segmentations and made further corrections, if necessary. Structural measures, including trabecular volume fraction (BV/TV), total cross-sectional area (Tt.Ar), total volume (Tt.Vol), cortical area (Ct.Ar), and the rectangular moments of inertia of area about the superior–inferior (*I*_SI_) and anterior–posterior (*I*_AP_) axes, were assessed from the 3D nano-CT volumes.

### PseudoDXA images

A 2D bone density map was generated for each FN VOI by projecting all bone voxels onto a planar FN ROI (pseudoDXA image). PseudoDXA images were created for cortical bone, trabecular bone, and cortical plus trabecular bone. Parameters calculated from the pseudoDXA images included the ROI area (pseudoDXA area), total number of bone voxels (pseudoDXA BMC), and total number of bone voxels/area (pseudoDXA aBMD). Average and minimum FN widths were measured directly from the pseudoDXA image. PseudoDXA images provided access to high-resolution structural measures and allowed an in-depth investigation of pseudoDXA parameters and structure and comparisons with experimentally determined strength.

### Mechanical testing

Whole-bone strength was measured directly by loading each proximal femur to failure in a fall-to-the-side configuration using a servohydraulic materials testing system (Instron 8511, Instron, Inc., Norwood, MA, USA), as described.^24^ Briefly, samples were oriented with the diaphyseal shaft at 10 degrees relative to a horizontal plane and the FN axis internally rotated 15 degrees, consistent with prior studies.^25^ Load was applied through a metal acetabular cup that was custom fit to the sample based on the femoral head diameter. Load was distributed to the greater trochanter using a custom polyester putty (Bondo, 3M, Inc, St. Paul, MN, USA)–filled pad. Bones were pre-loaded to 100N to ensure proper seating of the sample and fixtures and then loaded to failure at a displacement rate of 100 mm/s. Maximum load (N), also referred to as whole-bone strength, was calculated from the load–displacement curves. Data acquisition errors prevented the collection of strength data for two female and four male samples. The strength data included 35 female and 28 male samples reported previously.^20^^,^^26^

### Statistical analysis

Data were expressed as mean + SD and range and confirmed to be normally distributed based on the D'Agostino and Pearson test. Female and male donor traits were compared using independent *t*-tests. Given differences in the average age of female and male donors, each variable was adjusted to an age of 65 years. All linear regression analyses were conducted using the age-adjusted data.

### Sex-differences in the association between whole-bone strength and BMD

A linear regression analysis of age-adjusted strength vs age-adjusted pseudoDXA aBMD was conducted to test if our donor cohort replicated prior work. ANCOVA was used to test whether the slopes and elevations (*y*-intercepts) of the linear regressions differed between women and men. The strength difference between female and male donors was determined in the regression analysis by shifting the *x*-axis values of the female and male data by the mid-point across all data and then calculating the *y*-intercepts from the linear regression models. The *y*-intercepts of the *x*-shifted data reflect the elevations (ie, strength values) for females and males and allowed a determination of strength differences between sexes.

### Sex differences in the associations between strength and various size adjustments of pseudoDXA BMC

FN aBMD is calculated by dividing BMC by the projected area of the FN ROI. Work by others suggested dividing BMC by the projected area^1.5^ to account for out-of-plane thickness differences.^27^ Herein, we tested if differences in the strength–aBMD associations between sexes were affected by the morphological parameter used to size-adjust BMC. For this analysis, we examined linear regressions for strength vs pseudoDXA BMC, strength vs pseudoDXA BMC/FNW, strength vs pseudoDXA BMC/(pseudoDXA Area)^1.5^, and strength vs pseudoDXA BMC/Tt.Ar, where FNW = average FN width and Tt.Ar = average total cross-sectional area. All data were age-adjusted prior to conducting the linear regressions. Because the average Tt.Ar correlated highly with Tt.Vol, we report outcomes only for pseudoDXA BMC/Tt.Ar since the division of BMC by Tt.Ar and Tt.Vol outcomes were nearly identical. ANCOVA was used to test whether the slopes and elevations of the linear regressions differed between women and men. The strength difference between female and male donors was determined using the method described for the strength–aBMD association.

### Sex differences in the amount of bone relative to external size

The next analysis tested how pseudoDXA BMC and area correlated with structural traits using linear regression analysis. Since whole-bone strength is directly related to bone structure and the 3D distribution of bone tissue relative to the applied load, we tested whether the amount of tissue relative to total volume differed between sexes. To investigate why male FNs have a larger total volume for a given pseudoDXA area, we tested whether cross-sectional shape of the FN volume differed between sexes. For this, we tested if associations between measures of mass distribution and cross-sectional circularity and pseudoDXA area differed between sexes. Mass distribution measures included the moments of inertia about the superior–inferior (*I*_SI_) and anterior–posterior axes (*I*_AP_). The measure of circularity was calculated as the ratio of AP width to SI width, with a ratio of 1 indicating a circular cross-section. We also tested for sex differences in the associations between the amount of tissue relative to total volume for cortical and trabecular bone separately. Because BMC is used to estimate the relative amounts of cortical and trabecular bone,^28^ we investigated how pseudoDXA BMC related to the proportion of cortical and trabecular bone within the FN ROI and tested if these proportions differed between sexes.

### Associations between pseudoDXA parameters and strength

The relative contributions of various pseudoDXA parameters to strength were determined using a multivariable regression analysis with sex as a categorical variable (female = 0, male = 1). The goal of this analysis was to identify a set of traits that predicted strength without a significant sex effect. We examined different regression models to determine if adding more detailed structural information affected whether sex was a significant, independent contributor to strength. The final model included PYD (post-yield deflection), which was shown previously to contribute significantly to whole-bone strength for female proximal femurs.^20^ Analyses were conducted using GraphPad Prism (version 9.1.0, GraphPad Software, LLC, Boston, MA USA) or IBM SPSS Statistics (v 28.0.0; Armonk, NY USA). Significance was set at *P* < .05. Variance inflation factors (VIFs) were calculated for each model to test for collinearity among variables. If any model showed a variable with VIF > 10, the multivariable regression analysis would be repeated with the variable having the maximum VIF removed until all variables showed a VIF < 10. Two confirmatory analyses were conducted to test if the results were consistent when samples were limited to donors older than 60 years when testing for associations between strength and pseudoDXA aBMD and if the multivariable regression analysis results were explained by differences in body weight.

## Results

### Cohort characteristics

Donor ages spanned early adulthood to 89+ for both sexes (Table 1). On average, female donors were statistically significantly older than male donors (*P* = .043). Males had statistically significantly stronger proximal femurs and higher values for all pseudoDXA FN parameters including pseudoDXA aBMD, BMC, area, and the number of cortical and trabecular voxels (*P* < .001). However, the female FN region was constructed with a larger fraction of cortical bone relative to the total amount of bone compared to males (*P* = .032).

**Table 1:** Demographic, strength, and FN pseudoDXA measures.

Measure	Female	Male	*P*-value
Age (years)	67.8 ± 19.0 (24–97)	59.8 ± 19.1 (18–89)	.043
Strength (N)	3108.2 ± 1037.9 (1217.0–5264.9)	5230 ± 1482.2 (2432.6–8913.6)	.001
pseudoDXA area (cm^2^)	4.70 ± 0.26 (4.07–5.21)	5.43 ± 0.37 (4.60–6.16)	.001
pseudoDXA BMC (10^6^ bone voxels)	136.7 ± 31.3 (75.1–202.4)	198.3 ± 39.0 (109.2–259.7)	.001
pseudoDXA aBMD (10^6^ bone voxels/cm^2^)	29.1 ± 6.2 (16.7–41.9)	36.6 ± 7.0 (22.8–49.6)	.001
pseudoDXA BMC-cortical (10^6^ bone voxels)	80.1 ± 25.3 (34.6–138.5)	104.4 ± 23.1 (55.5–157.3)	.001
pseudoDXA BMC-trabecular (10^6^ bone voxels)	56.6 ± 14.0 (34.6–89.1)	93.8 ± 32.7 (36.9–176.0)	.001
% Bone voxels—cortical	57.7 ± 9.1 (37.2–72.1)	53.4 ± 10.0 (30.8–75.8)	.032

Data are unadjusted and shown as mean ± SD (range). Females: *n* = 51, except *n* = 49 for strength. Males: *n* = 44, except *n* = 40 for strength.

### Sex differences in the association between whole-bone strength and BMD

**Figure 1:**
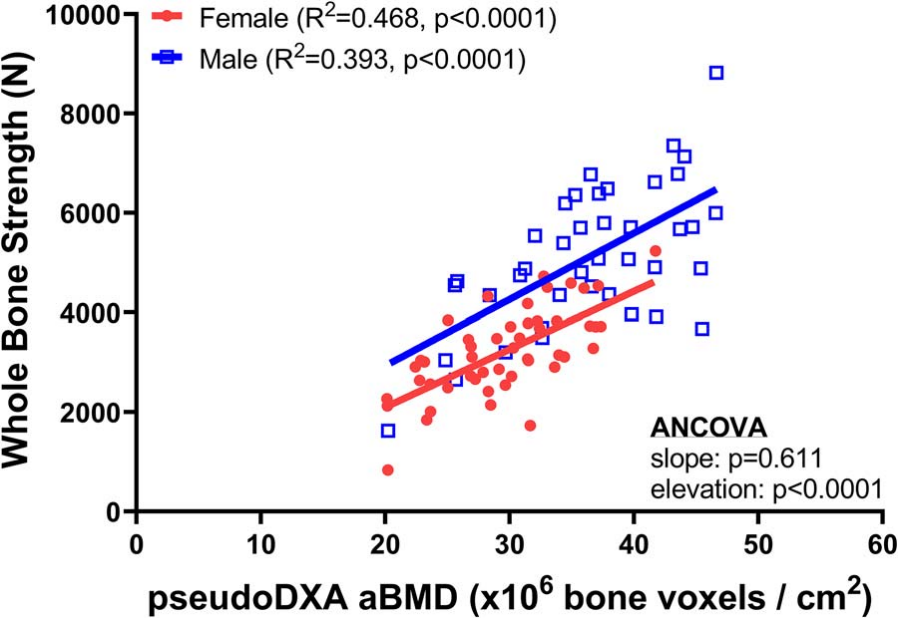
Whole-bone strength correlated significantly with pseudoDXA areal BMD (aBMD) for both sexes. Males showed a significantly greater elevation compared to females. Whole-bone strength and pseudoDXA aBMD data were adjusted to 65 years of age prior to conducting the linear regression analysis.

Whole-bone strength correlated statistically significantly with pseudoDXA aBMD (Figure 1) for females (*R*^2^ = 0.468, *P* < .0001) and males (*R*^2^ = 0.393, *P* < .0001). A comparison of the linear regressions showed no difference in the slope (*P* = 0.611, ANCOVA) but a significantly higher elevation (*y*-intercept) of the regression lines for males compared to females (*P* < .0001, ANCOVA). Male femurs were 25.6% (1045 N) stronger than female femurs, based on the difference in regression values calculated at the midpoint of the full range of pseudoDXA aBMD data. Similar outcomes were observed for the unadjusted data (Supplementary Table S1) and when limiting samples to donors older than 60 years (female *R*^2^ = 0.604, *P* < .0001; male *R*^2^ = 0.540, *P* < .0001; ANCOVA: slope, *P* = .243; elevation, *P* = .0004). The regression of strength vs body weight (Supplementary Figure S1) showed a significantly greater elevation for male proximal femurs compared to female (ANCOVA, *P* < .0001). Likewise, a significantly greater elevation was also observed for pseudoDXA aBMD vs body weight for men (ANCOVA, *P* = .006) (Supplementary Figure S2).

### Sex differences in the associations between strength and various size adjustments of pseudoDXA BMC

**Figure 2:**
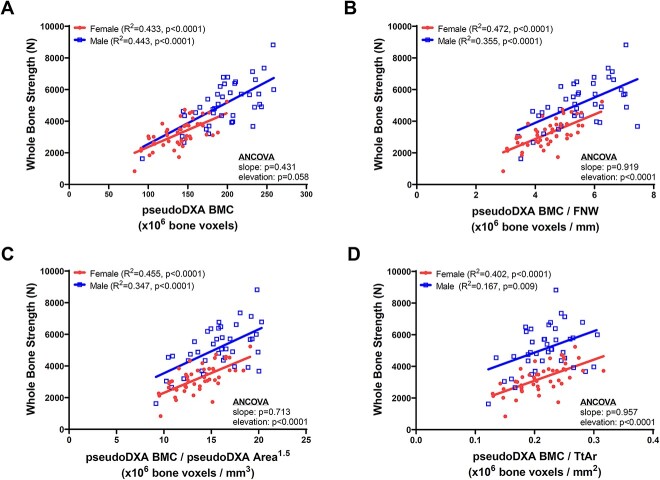
Linear regressions were conducted between whole-bone strength and (A) pseudoDXA BMC, (B) pseudoDXA BMC divided by FN width (FNW), (C) pseudoDXA BMC divided by pseudoDXA area raised to the 1.5 power, and (D) pseudoDXA BMC divided by the average total cross-sectional area (Tt.Ar) of the FN ROI. All data were adjusted to 65 years of age prior to conducting the linear regression analyses.

Whole-bone strength correlated significantly with pseudoDXA BMC (Figure 2A) for females (*R*^2^ = 0.433, *P* < .0001) and males (*R*^2^ = 0.443, *P* < .0001), and neither the slopes (*P* = .431) nor elevations (*P* = .058) differed significantly between sexes. Consistent linear regression outcomes were found when using unadjusted data (Supplementary Table S1) and when limiting the samples to donors older than 60 years (female *R*^2^ = 0.556, *P* < .0001; male *R*^2^ = 0.527, *P* < .0001; ANCOVA: slope, *P* = .399; elevation, *P* = .131). Linear regressions were conducted for pseudoDXA BMC divided by the average FN width (Figure 2B), pseudoDXA area^1.5^ (Figure 2C), and total cross-sectional area (Figure 2D). Strength correlated significantly with all size-adjusted pseudoDXA BMC measures for females and males. Each of the regressions between strength and BMC divided by a size measure showed no slope difference but did show significantly greater elevations for males compared to females (*P* < .0001, ANCOVA). When comparing elevations of the linear regressions, male femurs were 499N stronger than female femurs for pseudoDXA BMC; 1043N stronger for pseudoDXA BMC/FNW; 1361N stronger for pseudoDXA BMC/area^1.5^; and 1780N stronger for pseudoDXA BMC/TtAr (Table 2). Consistent linear regression outcomes were found when using unadjusted data (Supplementary Table S1).

**Table 2:** Strength values (N) and 95% CIs calculated at the midpoint of the female and male linear regressions.

Regression	Female	Male	Difference	% Difference
Strength vs BMC	3773 (3505–4040)	4272 (3808–4736)	499	12.4
Strength vs BMC/FNW	3586 (3368–3803)	4629 (4201–5058)	1043	25.4
Strength vs BMC/Area (aBMD)	3561 (3347–3775)	4606 (4190–5021)	1045	25.6
Strength vs BMC/Area^1.5^	3429 (3230–3629)	4790 (4390–5190)	1361	33.1
Strength vs BMC/TtAr	3262 (3065–3459)	5042 (4618–5466)	1780	42.9

*R*  ^2^ values for regressions are shown in Figure 2. Data were age adjusted to 65 years prior to the linear regressions. Percentage (%) indicates how the strength difference compared to the average strength of female and male proximal femurs combined.

### Sex differences in the amount of bone relative to external size

**Figure 3:**
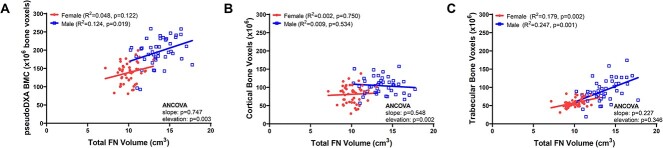
Linear regressions showing associations between (A) the total amount of bone, (B) the amount of cortical bone, and (C) the amount of trabecular bone relative to the total FN volume. All data were adjusted to 65 years of age prior to conducting the linear regression analyses.

Since whole-bone strength is derived from the three-dimensional (3D) distribution of bone tissue relative to the applied load, we tested if the amount of bone tissue relative to external size (total volume) differed between sexes. Males (*R*^2^ = 0.124, *P* = .019) but not females (*R*^2^ = 0.048, *P* = .122) showed a significant association between the total amount of bone (pseudoDXA BMC) and the total volume of the FN region (Figure 3A). Linear regression elevations for male FNs were significantly greater than for female FNs (*P* = .003, ANCOVA), indicating male FNs have more bone tissue for a given FN volume than females. Examining these associations for cortical and trabecular tissues separately, neither males (*R*^2^ = 0.009, *P* = .534) nor females (*R*^2^ = 0.002, *P* = .750) showed a significant association between the amount of cortical bone and total FN volume (Figure 3B). The different elevations (*P* = .002, ANCOVA) suggested males have more cortical bone within the total FN volume compared to women. Both males (*R*^2^ = 0.247, *P* = .001) and females (*R*^2^ = 0.179, *P* = .002) showed significant associations between the amount of trabecular bone and the total FN volume (Figure 3C). A comparison of the linear regressions showed no difference in the slopes (*P* = .227, ANCOVA) or elevations (*P* = .346, ANCOVA) indicating that both sexes showed similar associations between the amount of trabecular bone and total FN volume. Similar outcomes were found for the unadjusted data (Supplementary Table S1).

### Sex differences in the amount of bone relative to cross-sectional shape

**Figure 4:**
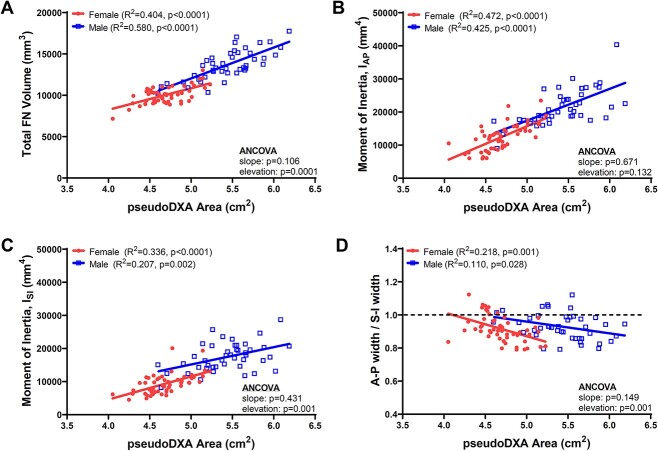
Linear regressions showing associations among (A) total FN volume and pseudoDXA area, (B) moment of inertia about the anterior–posterior axis (*I*_AP_) and pseudoDXA area, (C) moment of inertia about the superior–inferior axis (*I*_SI_) and pseudoDXA area, and (D) the ratio of FN widths measured in the anterior–posterior to superior–inferior directions relative to pseudoDXA area. All data were adjusted to 65 years of age prior to conducting the linear regression analyses.

Regressing total FN volume against pseudoDXA area showed significant correlations for women (*R*^2^ = 0.404, *P* < .0001) and men (*R*^2^ = 0.580, *P* < .0001) (Figure 4). The significant difference in the elevation indicated FN area reflects a larger total volume for men. Linear regressions of the moment of inertia calculated about the AP axis (*I*_AP_) vs pseudoDXA area were significant for women (*R*^2^ = 0.472, *P* < .0001) and men (*R*^2^ = 0.425, *P* < .0001) and without a sex-specific difference in the slope (*P* = .671, ANCOVA) or elevation (*P* = .132, ANCOVA). Men (*R*^2^ = 0.207, *P* = .001) and women (*R*^2^ = 0.336, *P* < .0001) showed significant associations between the moment of inertia calculated orthogonal to the plane of the DXA image (*I*_SI_). Unlike the association between *I*_AP_ and pseudoDXA area, differences in the elevation (*P* = .001, ANCOVA) for the linear regression of *I*_SI_ vs pseudoDXA area confirmed that men have a greater amount of bone distributed orthogonal to the DXA image. Both females (*R*^2^ = 0.218, *P* = .001) and males (*R*^2^ = 0.110, *P* = .028) showed a significant negative association between the average A-P width (orthogonal to DXA plane) divided by the S-I width (ie, FNW) (Figure 4D). The significant difference in elevations suggested that at similar values of pseudoDXA area, female proximal femurs have a more elliptical shape whereas male proximal femurs have a more circular shape for a given pseudoDXA area value.

### Sex differences in the amount of bone relative to the proportion of cortical and trabecular bone

**Figure 5:**
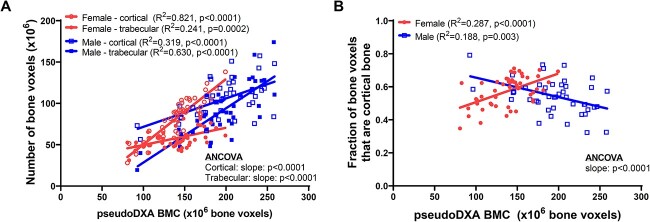
(A) Linear regressions showing different associations between the number of cortical (open circles, open squares) and trabecular (closed circles, closed squares) bone voxels relative to pseudoDXA BMC for female and male FNs. (B) The same data expressed as the ratio of cortical bone voxels relative to pseudoDXA BMC confirmed different regression slopes for female and male proximal femurs. All data were adjusted to 65 years of age prior to conducting the linear regression analyses.

PseudoDXA BMC was regressed against the amount of cortical bone and the amount of trabecular bone to test if variation in pseudoDXA BMC reflects different proportions of tissue types for women and men (Figure 5). The amount of cortical bone correlated significantly with pseudoDXA BMC for females (*R*^2^ = 0.821, *P* < .0001) and males (*R*^2^ = 0.319, *P* = .0001) (Figure 5A). Likewise, the amount of trabecular bone correlated significantly with pseudoDXA BMC for females (*R*^2^ = 0.241, *P* = .0002) and males (*R*^2^ = 0.630, *P* < .0001). However, significantly different slopes (*P* < .0001, ANCOVA) for both tissue types suggested that the variation in pseudoDXA BMC reflects different proportions of cortical and trabecular bone for women and men. A regression of the ratio of cortical bone to total bone versus pseudoDXA BMC confirmed that variation in BMC reflects different proportions of cortical and trabecular bone for women (*R*^2^ = 0.287, *P* < .0001) and men (*R*^2^ = 0.188, *P* = .003) (Figure 5B). A comparison of the regressions showed significantly different slopes (*P* < .0001, ANCOVA). Similar outcomes were found for the unadjusted data (Supplementary Table S1).

### Associations between pseudoDXA parameters and strength

The multivariable analysis aimed to identify a set of traits that predicted strength without a significant sex effect. The relative contributions of each pseudoDXA parameter to strength were determined using a multivariable regression analysis with sex as a categorical variable (Table 3). All variance inflation factors were <3, indicating the variables were not collinear. With each new regression, a new term was added or a bone mass term was replaced with new terms that were more descriptive of the bone mass distribution. The first model, which included sex, age, and pseudoDXA aBMD, confirmed the significant sex-specific effect reported thus far. The second model, which replaced aBMD with its constituents pseudoDXA BMC and pseudoDXA area, also showed a significant effect of sex. However, when replacing pseudoDXA BMC with measures of cortical and trabecular mass, the sex effect was no longer significant and remained non-significant even when adding PYD to the model. A confirmatory analysis was conducted by including body weight in the multivariable regression model to test if body weight accounted for the sex differences in the aBMD–strength association. This analysis was conducted for the sample subset of 35 female and 30 male donors with available body weight data. Strength and body weight did not differ significantly between groups with versus without body weight for either females or males (*P* > .5). Including body weight in the models did not alleviate the sex effect for any of the models (Supplementary Table S2).

**Table 3:** Multivariable linear regression analysis.

Variable	B (95% CI)	Standardized beta coeff	*P*-value	VIF
Model 1	Strength = constant + sex + age + pseudoDXA aBMD *R*_adj_^2^ =0.707 (0.001)			
**Sex**	**1066.8 (627.6–1506.0)**	**0.325**	**.001**	1.36
Age	–6.9 (−18.1 to 4.2)	−0.082	.219	1.31
**pseudoDXA aBMD**	**126.8 (94.7–158.9)**	**0.590**	**.001**	1.70
Model 2	Strength = constant + sex + age + pseudoDXA BMC + pseudoDXA area *R*_adj_^2^ =0.718 (0.001)			
**Sex**	**676.6 (53.7–1299.6)**	**0.206**	**.034**	2.85
Age	−6.7 (−17.6 to 4.2)	−0.079	.226	1.31
**pseudoDXA BMC**	**25.6 (19.3–31.8)**	**0.725**	**.001**	2.49
pseudoDXA Area	−309.9 (−948.6 to 328.9)	-0.090	.337	2.68
Model 3	Strength = constant + sex + age + cortical voxels + trabecular voxels + pseudoDXA area *R*_adj_^2^ =0.720 (0.001)			
Sex	605.8 (−23.2 to 1234.8)	0.185	.059	2.93
Age	−6.0 (−16.9 to 4.9)	−0.071	.279	1.32
pseudoDXA Area	−281.0 (−918.3 to 356.4)	−0.081	.383	2.70
**Cortical voxels**	**21.8 (13.4–30.2)**	**0.349**	**.001**	1.44
**Trabecular voxels**	**29.3 (21.0–37.7)**	**0.556**	**.001**	2.00
Model 4	Strength = constant + sex + age + cortical voxels + trabecular voxels + pseudoDXA area + PYD *R*_adj_^2^ = 0.743 (0.001)			
Sex	523.1 (−83.0 to 1129.3)	0.160	.090	2.96
Age	−9.0 (−19.7 to 1.7)	−0.106	.097	1.37
pseudoDXA Area	−283.0 (−894.4 to 328.3)	−0.082	.360	2.70
**Cortical voxels**	**21.4 (13.3–29.4)**	**0.342**	**.001**	1.45
**Trabecular voxels**	**28.7 (20.7–36.7)**	**0.544**	**.001**	2.01
**PYD**	**−82.9 (−140.3 to −25.4)**	**−0.161**	**.005**	1.08

Bold font indicates measures contributing significantly to strength. Abbreviation: VIF = variance inflation factor.

## Discussion

The goal of this study was to identify structural features contributing to differences in the strength–aBMD associations for women and men. Dividing BMC by any external size measure led to a significant sex-specific difference in the association between aBMD measures and strength. BMC alone predicted strength with only a minor sex-specific difference. aBMD values are influenced by bone size^27^^,^^29^ and men are generally thought to have higher aBMD than women because they have wider bones.^9^ Historically, dividing FN BMC by FN area was intended to minimize positioning errors and account for variation in BMC arising from bone size, body stature, and different imaging systems.^9^^,^^29^^,^^30^ However, our data indicate that the higher FN aBMD of men is not simply explained by differences in bone size between men and women. We identified three structural features that help explain why dividing FN BMC by a size measure led to sex-specific strength–aBMD associations: first, men have more bone mass (BMC) per unit volume than women; second, sex-specific differences in FN cross-sectional shape resulted in larger proportions of bone mass orthogonal to the plane of the DXA image in men compared to women; and third, men and women have different proportions of cortical and trabecular bone relative to BMC. These differences in the 3D shape of the FN and the internal distribution of cortical and trabecular tissues led to the greater strength of male proximal femurs relative to aBMD compared to females. Although body weight information was available for only 67% of the donor samples, the difference in the strength–aBMD association was not explained based on men being heavier on average than women (Supplementary Figures S1 and S2). This outcome was consistent with prior studies.^18^ Thus, our data suggest that the sex-specific discrepancy in the aBMD–strength association arises directly from the calculation of aBMD as BMC divided by a measure of bone size.

Whole-bone strength was measured directly in a simulated sideways-fall configuration, and strength values reported herein were consistent with prior studies.^2^^,^^7^^,^^8^^,^^31^ High-resolution nano-CT image volumes were used to generate pseudoDXA images, which have been previously validated against clinical-type DXA images.^20^ Male proximal femurs were significantly stronger for a given pseudoDXA aBMD compared to female proximal femurs, consistent with prior work reported for proximal femurs imaged using clinical DXA systems.^5–8^ Male femurs were 25.6% (1045 N) stronger than female femurs for a given pseudoDXA aBMD, which was considered quite large. Thus, a major outcome of this study was independently replicating sex-specific associations between proximal femur strength and FN aBMD.

Three structural features were identified that help explain why dividing FN BMC by a bone size measure led to sex-specific strength–aBMD associations. First, female and male proximal femurs showed different associations between pseudoDXA BMC and area. Even with overlapping FN areas for men and women, men showed a greater amount of bone contained within a larger FN volume and projected area compared to women. This outcome was expected and consistent with prior work.^6^ Although men have wider bones than women, on average, men also have a proportionally greater amount of bone tissue contained within this volume independent of body weight^16^ and external bone size.^17^ Segregating FN BMC into cortical and trabecular tissues revealed that the sex-specific difference in the amount of bone relative to volume arose largely from cortical bone and not trabecular bone. The total amount of bone incorporated into the FN was largely independent of external volume for women, consistent with prior work,^32^ but was significant for men, which was not examined previously. Taken together, our data suggest that FNs are constructed with similar amounts of cortical bone across the range of external bone sizes but with the amount of trabecular bone being different and proportional to external size. The greater cortical mass of men than women may reflect differences in growth patterns and body stature.^33^ Similar associations between cortical bone and external size were reported by others and help explain why cortical thickness is greater in narrow compared to wider bones.^32^

**Figure 6:**
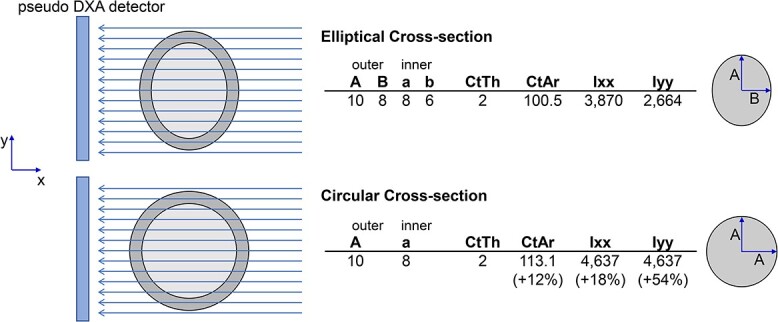
The impact of different cross-sectional shapes on a DXA image is illustrated in an example of two idealized (cylindrical) FN structures of length *L*. The two structures have the same FN width (FNW) and cortical thickness (CtTh) and consequently have the same projected areas. Because the two cylinders have different cross-sectional shapes, the elliptical structure will have a smaller mass (Ct.Ar) compared to the circular structure. BMC is calculated as CtAr × *L*, so differences in CtAr reflect differences in BMC. The moments of inertia about the horizontal plane (Ixx) and vertical plane (Iyy) are shown to convey the greater strength of the circular structure in different loading directions. For this example, the 12% gain in BMC of the circular structure translated to an 18% increase in strength about the *x*-axis and a 54% increase in strength about the *y*-axis.

The second structural feature that may contribute to different aBMD values between men and women was that sex-specific differences in FN shape resulted from greater out-of-plane width, which was reflected in men having a more circular cross-sectional shape for a given pseudoDXA area compared to women. The negative correlation in Figure 4D indicates larger (wider) FNs have a more elliptical shape, whereas smaller (narrower) FNs have a more circular shape. Cross-sectional shape circularity results from an FN having more tissue orthogonal to the plane of the DXA image compared to an elliptical shape. This outcome is consistent with prior work examining variation in FN shape.^34^ Differences in shape relative to external size have also been reported for the metacarpal.^26^ When comparing a circular and elliptical cross-sectional shape with the same FNW and cortical thickness, the more circular shape projects more bone onto the DXA image, thereby resulting in a larger BMC relative to the projected area (Figure 6). When holding all else constant, pseudoDXA area will reflect a greater proportion of out-of-plane bone tissue for narrower, more circular FNs and less for wider, more elliptical FNs. Although the impact of this shape difference on fracture resistance is not clear, we speculate that a more circular cross-section may increase fracture resistance for loads applied at a greater range of angles and thereby reduce the likelihood of transitioning a fall into a hip fracture.

The third structural feature that may contribute to different aBMD values was finding that pseudoDXA BMC reflected different proportions of cortical and trabecular bone for men and women. This association, which is a new finding, also varied within each sex. Prior studies estimating bone structure from DXA FN images assumed that BMC reflects a fixed ratio of cortical to trabecular mass of 0.6.^28^ Our data showed that this ratio is not fixed but varies significantly with external size and differs between men and women. Figure 5 shows that the proportion of cortical bone is greatest at lower values of pseudoDXA BMC for males but is greatest at higher values of pseudoDXA BMC for females. How the dependence of the proportion of cortical and trabecular on external size affects estimates of FN geometry^28^ has yet to be determined. Further, longitudinal studies are needed to determine if the associations shown in Figure 5A reflect how the proportions of tissue types change over time commensurate with age-related declines in BMC.

Bones fracture when applied loads exceed the strength or load-carrying capacity.^35^ Bone strength cannot be measured directly in situ; consequently, non-invasive technologies are needed to estimate strength and fracture resistance.^36^ Although aBMD assesses bone mass but not strength per se, low aBMD is associated with increased fracture risk^37–39^ and is generally thought to reflect low strength.^40^ Understanding discrepancies in the aBMD–strength association is important to ensure information arising from a standard DXA scan informs clinicians on how best to treat individuals. aBMD values for men and women are generally not compared directly except when a sample of young adult women is used as the reference population to calculate T-scores for men. Men have stronger bones than women to accommodate, in part, their greater body weight, and this is reflected in men having higher aBMD than women, on average. Critically, the greater strength of men was not simply proportional to the difference in aBMD but also included an offset that was equivalent to an additional ~25% increase in strength compared to women. Importantly, clinical aBMD and T-scores are not adjusted for body weight. Thus, T-scores for men, when calculated using a female reference population, may not accurately reflect strength because this ~25% discrepancy is not considered. Men have a higher fracture-related mortality than women,^41^ which means accurately diagnosing men who would benefit from prophylactic treatment is particularly critical. Further, sex-based reference populations may not be informative for intersex^42^ and transgender individuals^43^ who may be better supported clinically by having a predictor of bone strength without a sex-specific discrepancy.^43^

There are several limitations with this study that are worth discussing. First, the cadaveric sample was comprised of White female and male donors. We focused initially on this population because of the higher fracture incidence of White women and men,^44^ but recognize the importance of extending these investigations to other races/ethnicities^45^ and genders.^42^^,^^43^ How inter-individual and inter-population differences in structure affect the association between aBMD and strength has not been well studied. Investigating these discrepancies is important given the variability in how T-scores and fracture-risk indices are adjusted for race/ethnicity.^46^ The number of individuals that do not self-identify with discrete racial/ethnic categories is growing rapidly.^47^ These individuals would benefit from having diagnostic tools that reflect low bone strength without racial/ethnic discrepancies. Second, the FN ROI examined in this study was based on the Hologic DXA system to be consistent with the validation study.^20^ Examining other ROIs is warranted to determine if the sex-specific differences reported herein depend on the size and anatomical location of the ROI. It is important to note that prior studies reporting a sex difference used the Lunar DXA system,^5–8^ and so, we would expect the sex difference to hold across FN ROI locations. Third, pseudoDXA images are idealized as they provide bone voxel information at high resolution and segmented for cortical and trabecular tissues, but without attenuation features like scattering and imaging variation due to differences in detectors or resolution. Nevertheless, the pseudoDXA images allowed us to investigate the strength–aBMD association by providing access to 3D structural information that is not readily available from traditional 2D DXA images.

In conclusion, dividing BMC by a measure of bone size contributed to sex-specific differences in the association between aBMD and proximal femur strength. aBMD is widely used clinically for monitoring individuals for osteoporosis and as an indication of low bone strength and increased fracture risk. The calculation of T-scores, which compares a person’s aBMD to that of a reference population, is central to establishing treatment options. Our analyses showed that the proximal femurs of women were not simply smaller (narrower) versions of the proximal femurs of men but are constructed in fundamentally different manners.^7^ The more circular cross-sectional morphology and greater amount of cortical bone relative to external size of male compared to female FNs preclude an equitable adjustment of BMC by any measure of bone size. Given the prevalent clinical use of aBMD to identify individuals at risk of fracturing and for monitoring the effects of treatment, our data provide new insight into why a new approach for incorporating differences in bone size and body stature into measures of bone mass assessed by DXA is warranted. We suggest that further establishing differences in associations between bone strength and structure provides a new research direction and will enable investigations to determine how differences in DXA images relate to clinical diagnoses and treatment strategies across heterogeneous populations.

## Supplementary Material

Fig_S1-Max_vs_BW_MvF_ziae013

Fig_S2-aBMD_vs_BW_MvF_ziae013

Supplementary_Table_S1

Supplementary_Table_S2

## Data Availability

The nano-CT image volumes are available for use for research purposes. The fully convolutional neural networks used for segmentation are available for use. Excel spreadsheets with raw data are available for use.
