# Supplementary figures and images for: Structural differences contributing to sex-specific associations between FN BMD and whole-bone strength for adult White women and men
Source: JBMR Plus. 2024 Jan 30;8(4):ziae013. doi: 10.1093/jbmrpl/ziae013 (PMC10958990; doi:10.1093/jbmrpl/ziae013)

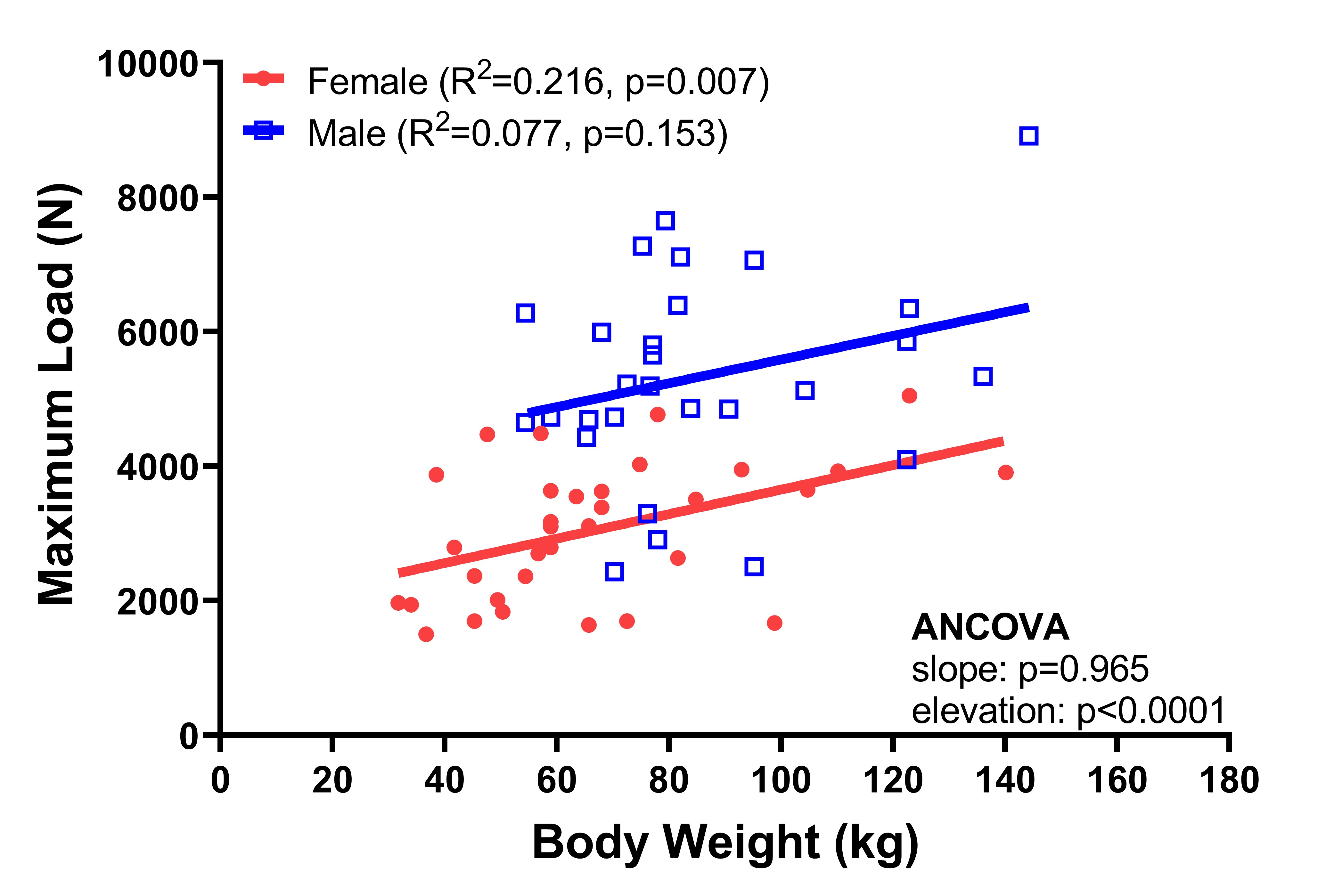

Supplement: Fig_S1-Max_vs_BW_MvF_ziae013 [file fig_s1-max_vs_bw_mvf_ziae013.jpeg]

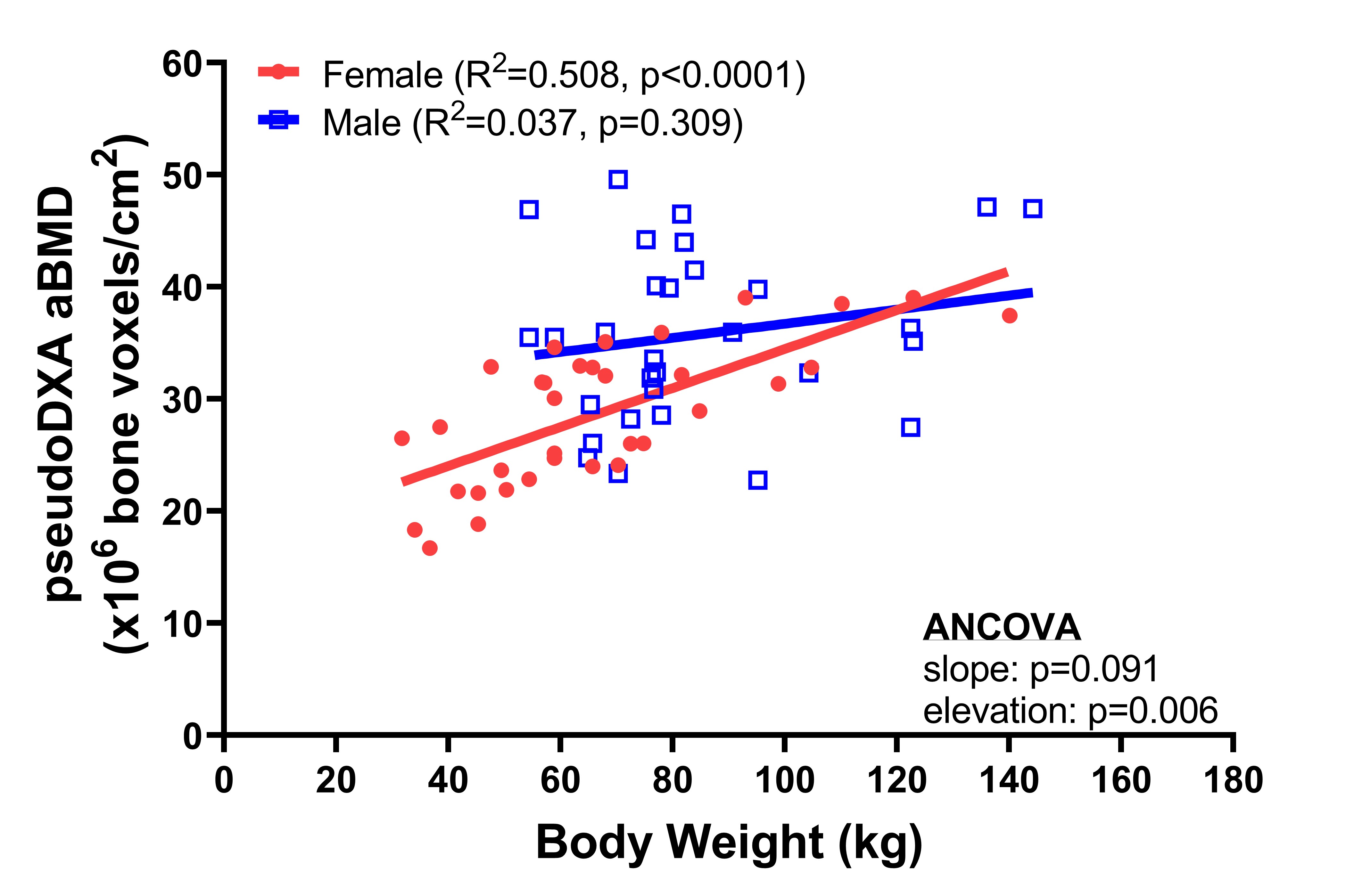

Supplement: Fig_S2-aBMD_vs_BW_MvF_ziae013 [file fig_s2-abmd_vs_bw_mvf_ziae013.jpeg]
