# Supplementary material for: Structural differences contributing to sex-specific associations between FN BMD and whole-bone strength for adult White women and men
Source: JBMR Plus. 2024 Jan 30;8(4):ziae013. doi: 10.1093/jbmrpl/ziae013 (PMC10958990; doi:10.1093/jbmrpl/ziae013)
Supplement: Supplementary_Table_S1 [file supplementary_table_s1.pdf]

Table S1. Linear regression outcomes using unadjusted data

Comparison	Female – R^2 (p-value)	Male – R^2 (p-value)	ANCOVA: slope	ANCOVA: elevation
Strength vs pseudoDXA aBMD	0.609 (0.0001)	0.441 (0.0001)	0.767	0.0001
Strength vs pseudoDXA BMC	0.568 (0.0001)	0.494 (0.0001)	0.693	0.078
Strength vs pseudoDXA BMC/FNW	0.608 (0.0001)	0.442 (0.0001)	0.773	0.0001
Strength vs pseudoDXA BMC/TtAr	0.543 (0.0001)	0.213 (0.003)	0.817	0.0001
Strength vs pseudoDXA BMC/Area^1.5	0.597 (0.0001)	0.384 (0.0001)	0.907	0.0001
pseudoDXA BMC vs Total FN Volume	0.0195 (0.328)	0.102 (0.035)	0.610	0.005
Cortical bone voxels vs Total FN Volume	0.0001 (0.933)	0.009 (0.545)	0.701	0.001
Trabecular bone voxels vs Total FN Volume	0.084 (0.039)	0.199 (0.002)	0.233	0.079
pseudoDXA BMC vs pseudoDXA Area	0.190 (0.001)	0.067 (0.090)	0.272	0.001
Cortical bone voxels vs pseudoDXA Area	0.196 (0.001)	0.057 (0.119)	0.075	0.424
Trabecular bone voxels vs pseudoDXA Area	0.031 (0.219)	0.019 (0.367)	0.863	0.0003
Total FN Volume vs pseudoDXA Area	0.411 (0.0001)	0.581 (0.0001)	0.107	0.001
Ixx vs pseudoDXA Area	0.439 (0.0001)	0.426 (0.0001)	0.797	0.107
Iyy vs pseudoDXA Area	0.283 (0.0001)	0.204 (0.002)	0.553	0.0002
AP width/SI width vs pseudoDXA Area	0.221 (0.0005)	0.107 (0.030)	0.141	0.0006
Cortical bone voxels vs pseudoDXA BMC	0.807 (0.0001)	0.297 (0.0001)	0.0001	n/a
Trabecular bone voxels vs pseudoDXA BMC	0.375 (0.0001)	0.649 (0.0001)	0.0001	n/a
Fraction of cortical voxels vs pseudoDXA BMC	0.181 (0.002)	0.159 (0.007)	0.0001	n/a

n/a: ANCOVA results report p-values for slope and elevation, but elevation only if the slope is not significantly different.
