# Supplementary material for: Structural differences contributing to sex-specific associations between FN BMD and whole-bone strength for adult White women and men
Source: JBMR Plus. 2024 Jan 30;8(4):ziae013. doi: 10.1093/jbmrpl/ziae013 (PMC10958990; doi:10.1093/jbmrpl/ziae013)
Supplement: Supplementary_Table_S2 [file supplementary_table_s2.pdf]

Table S2. Multivariable linear regression analysis including body weight

Variable	B (95% CI)	Standardized Beta Coeff	p-value	VIF
Model 1	Strength = constant + sex + age + pseudoDXA aBMD + body weight R_{adj}^2 =0.718 (0.001)			
**Sex**	1291.4 (755.5 - 1827.4)	**0.380**	**0.001**	1.32
Age	-4.5 (-17.9 - 8.8)	-0.056	0.500	1.44
**pseudoDXA aBMD**	**120.9 (77.4 - 164.5)**	**0.554**	**0.001**	2.11
Body weight	2.6 (-7.9 - 13.2)	0.041	0.503	1.42
Model 2	Strength = constant + sex + age + pseudoDXA BMC + pseudoDXA area + body weight. R_{adj}^2 =0.729 (0.001)			
**Sex**	**874.7 (68.8 - 1680.6)**	**0.258**	**0.034**	3.10
Age	-3.8 (-16.9 - 9.3)	-0.047	0.563	1.44
**pseudoDXA BMC**	**24.8 (16.4 – 33.2)**	**0.692**	**0.001**	3.02
pseudoDXA Area	-281.5 (-1082.7 - 519.7)	-0.082	0.484	2.99
Body weight	2.1 (-8.3 - 12.4)	0.032	0.690	1.43
Model 3	Strength = constant + sex + age + + (cortical voxels + trabecular voxels) + pseudoDXA area + body weight. R_{adj}^2 =0.724 (0.001)			
**Sex**	860.9 (30.8 - 1691.102)	**0.254**	**0.042**	3.23
Age	-3.9 (-17.1 - 9.4)	-0.048	0.560	1.45
pseudoDXA Area	-271.3 (-1089.4 546.8)	-0.079	0.509	3.06
**Cortical voxels**	**24.0 (10.9 – 37.1)**	**0.319**	**0.001**	1.63
**Trabecular voxels**	**25.3 (15.1 - 35.4)**	**0.510**	**0.001**	2.27
Body weight	2.1 (-8.4 - 12.5)	0.032	0.694	1.43
Model 4	Strength = constant + sex + age + + (cortical voxels + trabecular voxels) + pseudoDXA area + PYD + body weight. R_{adj}^2 =0.747 (0.001)			
**Sex**	**934.8 (137.9 - 1731.8)**	**0.275**	**0.022**	3.25
Age	-10.1 (-23.8 - 3.6)	-0.125	0.144	1.69
pseudoDXA Area	-454.4 (-1251.7 – 343.0)	-0.132	0.258	3.17
**Cortical voxels**	**22.9 (10.4 – 35.5)**	**0.305**	**0.001**	1.63
**Trabecular voxels**	**23.6 (13.8 - 33.5)**	**0.477**	**0.001**	2.32
**PYD**	**-98.5 (-179.2 - -17.7)**	**-0.178**	**0.018**	1.26
Body weight	3.7 (-6.4 - 13.8)	0.058	0.462	1.46

Bold font indicates measures contributing significantly to strength; VIF = Variance Inflation Factors
